# FACTORS INFLUENCING WHERE AFRICAN IMMIGRANTS PLAN TO SEEK CARE FOR A RELATIVE WITH DEMENTIA

**DOI:** 10.1093/geroni/igac059.028

**Published:** 2022-12-20

**Authors:** Manka Nkimbeng, Wynfred Russel, Tetyana Shippee, Joseph Gaugler

**Affiliations:** University of Minnesota, Minneapolis, Minnesota, United States; African Career Education And Resources Inc. (ACER), Brooklyn Park, Minnesota, United States; University of Minnesota, Minneapolis, Minnesota, United States; University of Minnesota, Minneapolis, Minnesota, United States

## Abstract

Many African immigrants did not hear about dementia until they migrated to the United States, which limits understanding of the disease and awareness of community resources. We examined the relationship between being a caregiver, care recipient country of residence, and seeking care using data from 152 African immigrants. Most participants (90%) were caregivers, 12% of the care recipients resided in Africa, and 59% reported that they would seek care within the formal health system. After controlling for age, gender, income and participant place of birth, caregivers were more likely (aOR: 2.31, 95% CI 0.37, 13.39) to seek care from community networks alone, as were those providing care to a recipient in Africa (aOR: 1.94, 95% CI 0.56, 6.71), but these were not statistically significant. These findings which trend toward preferences for seeking care through informal community networks (friends, religious organizations etc.), can inform dementia education and outreach for this community.

